# Brain Region Differences in α1- and α5-Subunit-Containing GABA_A_ Receptor Proteomes Revealed with Affinity Purification and Blue Native PAGE Proteomics

**DOI:** 10.3390/cells13010014

**Published:** 2023-12-20

**Authors:** Miao Chen, Frank Koopmans, Miguel A. Gonzalez-Lozano, August B. Smit, Ka Wan Li

**Affiliations:** Department of Molecular and Cellular Neurobiology, Center for Neurogenomics and Cognitive Research, Amsterdam Neuroscience, Vrije Universiteit Amsterdam, 1081 HV Amsterdam, The Netherlands; miao.chen@vu.nl (M.C.); miguel_gonzalezlozano@hms.harvard.edu (M.A.G.-L.); guus.smit@vu.nl (A.B.S.)

**Keywords:** GABA_A_ receptor, immunoprecipitation, proteomics, brain, synapse, immunocytochemistry

## Abstract

GABA_A_ receptors are the major inhibitory receptors in the brain. They are hetero-pentamers with a composition of predominantly two α, two β, and one γ or δ subunit. Of the six α subunit genes, the α5 subunit displays a limited spatial expression pattern and is known to mediate both phasic and tonic inhibition. In this study, using immunoaffinity-based proteomics, we identified the α5 subunit containing receptor complexes in the hippocampus and olfactory bulb. The α1–α5 interaction was identified in both brain regions, albeit with significantly different stoichiometries. In line with this, reverse IPs using anti-α1 antibodies showed the α5–α1 co-occurrence and validated the quantitative difference. In addition, we showed that the association of Neuroligin 2 with α1-containing receptors was much higher in the olfactory bulb than in the hippocampus, which was confirmed using blue native gel electrophoresis and quantitative mass spectrometry. Finally, immunocytochemical staining revealed a co-localization of α1 and α5 subunits in the post-synaptic puncta in the hippocampus.

## 1. Introduction

GABA_A_ receptors (GABA_A_Rs) are pentameric ligand-gated chloride ion channels that mediate major inhibitory neurotransmission in the central nervous system. There are 19 known GABA_A_ receptor subunits, α1-6, β1-3, γ1-3, δ, ε, π, ρ1-3, and θ [1]. Synaptic GABA_A_ receptors are predominantly assembled from two copies of α1, α2, or α3, two copies of β1, β2, or β3, and a single copy of γ2, whereas the extra-synaptic GABA_A_ receptors contain two copies of α4, α5, or α6, two copies of β, and often a single copy of δ [2]. The specific subunit compositions of these receptors underlie their distinct electrophysiological and pharmacological properties [3,4]. Synaptic GABA_A_ receptors mediate phasic inhibition, whereas tonic inhibition is mediated by extra-synaptic GABA_A_ receptors [5]. The expression of the subunits shows considerable heterogeneity across the brain, implicating brain region differences in the formation of GABA_A_R subtypes, some of which have been targeted as druggable candidates for the treatment of brain disorders [4]. However, the GABA_A_ receptor subunit composition and the receptor-associated proteins in various brain regions have not been examined in depth.

GABA_A_-receptor-associated proteins are known to be involved in the formation, trafficking, localization, and modulation of the channel properties of the receptors [6,7]. For instance, the scaffolding protein Gephyrin (Gphn) is located in the GABAergic synapses. It interacts with GABA_A_ receptor subtypes containing one of the α1-3 subunits and the γ2 subunit and is reported to be involved in GABA_A_ receptor synaptic clustering [7,8,9]. Neuroligin 2 (Nlgn2) and Lipoma HMGIC fusion partner-like 4 (Lhfpl4) have been found to regulate the formation of an inhibitory synapse and the maintenance of GABA_A_ receptor clustering at inhibitory synaptic sites [10,11,12,13,14].

The α5-containing GABA_A_ receptor represents about 5% of the total GABA_A_R population in the brain, with the highest expression in two regions, the hippocampus and olfactory bulb. In the hippocampus, over 25% of neurons express the α5 gene [15,16], in particular in the hippocampal CA1 and CA3 regions involved in hippocampus-dependent cognitive tasks, such as learning and memory formation [17,18]. In the olfactory bulb, >30% of internal granule neurons contain α5 GABA_A_ receptors, but the function of this receptor type in the olfactory bulb remains unclear [19].

The α5-containing GABA_A_ receptors have a restricted brain expression pattern but are known to play important roles in cognitive processes, such as learning and memory formation [20,21,22]. Deficits in α5 GABA_A_ receptor expression levels also have been observed in patients with brain disorders, e.g., autism spectrum disorder [23], Alzheimer’s disease [24], intellectual disabilities, and epilepsy [25]. Immunofluorescence staining revealed the α5 GABA_A_ receptor to be largely extrasynaptically localized. Activated radixin has been identified as a direct interaction partner to α5-containing GABA_A_ receptors, facilitating receptor binding to cytoskeletal elements [26], receptor clustering [27], and recruitment [28]. The α5-subunit-containing receptor predominantly mediates tonic inhibition, especially in the hippocampal CA1 region [15,29]. During circuit development in the first 3 weeks, α5-containing GABA_A_ receptors are also found to cluster at synaptic sites where they interact with Gphn and mediate phasic inhibition [18,27,30,31,32].

In the present study, we applied a previously reported interaction–proteomics research platform on hippocampus and olfactory bulb extraction [33] to reveal the α5 GABA_A_ receptor composition and found substantial heterogeneity of α5-containing receptor subtypes. In particular, the receptors with α1α5 subunits in the hippocampus are more abundant than in the olfactory bulb. Immunocytochemical staining of α1 and α5 subunits revealed synaptic localization of this receptor subtype on cultured hippocampal neurons. Moreover, we found that the Nlgn2 and α1 interaction was higher in the olfactory bulb than in the hippocampus. Blue native gel electrophoresis in conjunction with mass spectrometry confirmed this difference in the interaction pattern of Nlgn2 to α1β2γ2 GABA_A_Rs in the hippocampus and olfactory bulb.

## 2. Materials and Methods

### 2.1. Animals and Brain Material

C57BI/6J mice were bred in our facility. Adult mice were sacrificed by decapitation at 6 months of age, the hippocampus and olfactory bulb were separately dissected and labeled, and the dissected tissues were stored at −80 °C until further use. The Animal Users Care Committee of the Vrije Universiteit Amsterdam gave permission for all experiments, which were performed according to the relevant guidelines and regulations.

### 2.2. Immunoprecipitation and SDS-PAGE

Immunoprecipitation and SDS-PAGE were similarly prepared as previously described [33,34]. In short, mouse hippocampi and olfactory bulbs were dissected and homogenized in 1% maltose-neopentyl glycol (MNG) buffer (25 mm HEPES, 150 mm NaCl (pH 7.4), and proteinase inhibitor (Roche)) using a homogenizer (Sartorius, Göttingen, Germany; 12 strokes, 900 rpm). The homogenate was incubated for 1 h at 4 °C and then centrifugated twice at 20,000× *g* for 30 min. The supernatant was collected and equally aliquoted for immunoprecipitation. Two-microgram antibodies were added for each IP replicate, and the mixture of supernatants and antibodies was incubated overnight on a rotator at 4 °C. Then, 40 μL of protein A/G plus agarose beads (Santa Cruz, Dallas, TX, USA) were added and washed four times with a 0.1% MNG wash buffer with 25 mm HEPES and 150 mm NaCl (pH 7.4). A 2X SDS sample buffer was used to elude the proteins bound on the beads. Samples were heated to 98 °C for 5 min and ran on a 10% SDS polyacrylamide gel and stained with Coomassie Blue. Stained protein bands were cut from the gel and transferred to a 96-well plate (0.45 μm filter; MultiScreen-HV 96-well filter plate from Millipore, Burlington, MA, USA). Gel pieces were destained, digested with trypsin, and dried using SpeedVac (Eppendorf, Hamburg, Germany) as previously described.

The antibodies used were from NeuroMab, Davis, CA, USA. The α1 (Gabra1) (N95/35, AB_106697873), α5 (Gabra5) (N52A/42, AB_2491075), and Nlgn2 antibodies were from Synaptic Systems (SySy) (129 511, Göttingen, Germany). We obtained custom-made antibodies from Genscript against GluA2/3 (sequence: QNFATYKEGYNVYGIESVKI).

### 2.3. Blue Native-PAGE (BN-PAGE)

BN-PAGE was performed according to a previously described approach [35,36]. In brief, the dissected mouse hippocampus and olfactory bulb were homogenized with 1% MNG extraction buffer, and the supernatant was collected after centrifugation. An amount of 10 µg of solubilized protein samples were mixed with BN loading buffer, molecular weight markers (Thermo Fisher, Waltham, MA, USA), and G-250 Coomassie Blue at appropriate ratios according to the manual. The sample mixture was loaded on a pre-cast Invitrogen NativePAGE 4–16% Bis–Tris Gel (Thermo Fisher, Waltham, MA, USA). The gel was run at a 2.5 h setting (running condition: 150 V for 1.5 h at 4 °C, followed by 250 V for 1 h at 4 °C).

For mass spectrometry, the gel after the running was fixed overnight in a fresh fixation buffer containing 200 mL of 50% ethanol, 7% acetic acid, and 3% phosphoric acid. The gel was washed three times on the second day in water until protein bands were visible. A grid cutter (The Gel Company, San Francisco, CA, USA) was used to cut each sample line into 50 equally sized pieces and individually transferred into wells on a 96-well filter plate. The proteins in gel pieces were reduced by treating with 100 µL Tris(2-carboxyethyl) phosphine hydrochloride at 37 °C for 30 min, followed by treating with methyl methanethiosulfonate. For each step, a brief wash with water and centrifugation of the plate were performed to remove the supernatant. The sample preparation for mass spectrometry was performed as described previously [33].

For immunoblotting, native hippocampal and olfactory bulb lysates were run on the pre-cast NativePAGE gel using the same running condition. Proteins were transferred onto a PVDF membrane overnight at 4 °C. After the transfer, the proteins on the membrane were fixed using a membrane fixation buffer (8% acetic acid) for 30 min. Western blotting was performed as described previously [37]. The primary antibodies were Anti-Gabra1 (NeuroMab, Davis, CA, USA, N95/35, AB_106697873, 1:1000) and Anti-Gabrb2/3 (NeuroMab, Davis, CA, USA, 62-3G1, AB_2315837, 1:1000). The secondary antibody was HRP-conjugated Goat anti-Rabbit IgG (Dako, Santa Clara, CA, USA, 1:1000).

### 2.4. HPLC-ESI MS/MS

The mass spectrometry setup used for analyzing the immunoprecipitation and BN-PAGE-MS samples was previously described [38]. In brief, the peptide samples were prepared using Evotip and run on a 15 cm × 75 µm, 1.9 µm Performance Column (EV1112 from EvoSep, Odense, Danmark) with the 30-samples-per-day program. Peptides were electro-sprayed into a TimsTOF Pro 2 mass spectrometer (Bruker, Billerica, MA, USA) and analyzed with diaPASEF [39]. The MS scan was between 100 and 1700 *m*/*z*. The Tims settings were 1/Ko from start to end and between 0.6 and 1.6 V.s/cm^2^, a ramp time of 100 ms, an accumulation time of 100 ms, and a ramp rate of 9.42 Hz.

### 2.5. Data Analysis

DIA-PASEF raw data were processed with DIA-NN 1.8 [40]. An in silico spectral library was generated from the uniprot mouse proteome (SwissProt and TrEMBL canonical sequences, released 2019-10) using Trypsin/P digestion and at most 1 missed cleavage. The peptide length range was set to 7–30. The precursor charge range was set to 2–4. The precursor *m*/*z* range was set to 300–1400. The fragment ion *m*/*z* range was set to 200–1800. The maximum number of variable modifications was set to 1. The Precursor False Discovery Rate (FDR) was 1% (default). Both the MS1 and MS2 mass accuracies were set to 10 ppm, and double-pass mode was enabled. Protein inference was set to isoform. All other settings were left as default.

MS-DAP 1.0.5 [41] was used to preprocess the DIA-NN results, and the subsequent analyses described here were implemented in R. “VWMB” peptide-level normalization was used to reduce variation among replicates, and subsequently, “modebetween_protein” was used to balance the between-experimental-condition abundance levels at the protein level. From the MS-DAP peptide-level data table, we selected peptides for proteins of interest and used these to compute iBAQ pseudo-absolute abundance values per protein by dividing their protein sum intensities (of respective peptides, per sample) by the number of theoretically observable peptides per protein. To compare relative prey protein abundances of pulldowns against the same bait between brain regions, we first normalized their iBAQ abundance values per sample to the respective bait protein’s abundance. Linear regression was applied to these data to test whether the change in relative prey abundance between brain regions was significant, with ‘datasets’ as a covariate to account for our independent replicates performed across 2 cohorts. Peptides shared among subunits were excluded as they are not informative for the specific subtypes of the receptor.

### 2.6. Immunocytochemistry

The hippocampi obtained from E18 wildtype mice (C57Bl6 mice, Vrije Universiteit Amsterdam) underwent dissection and were then immersed in Hank’s balanced salts solution (HBSS) (1 M, Thermo Fisher, Waltham, MA, USA). The hippocampi were digested in a buffer containing 7 mM HEPES at pH 7.4 and 0.25% trypsin (Thermo Fisher, Waltham, MA, USA). Afterwards, the hippocampi underwent triple washing in HEPES buffer and double washing in neurobasal medium (Thermo Fisher, Waltham, MA, USA). Subsequently, neurons derived from the hippocampi were mechanically dissociated using Pasteur pipettes, and the neuronal cell density was determined using a Fuchs–Rosenthal chamber. An amount of 12.5 K cells/well were plated for culturing in neurobasal medium supplemented with 2% B-27 (Thermo Fisher, Waltham, MA, USA), 2% HEPES solution (pH 7.4), 0.25% glutamine (200 mM, Thermo Fisher, Waltham, MA, USA), and 1% penicillin/streptomycin on a 96-well glass-plate bottom, which was coated with poly-D-lysine/laminin (Sigma-Aldrich, St. Louis, MO, USA) and then incubated at 37 °C with 5% CO_2_. Neurons at DIV7 were fixed with 4% paraformaldehyde and 1% sucrose in phosphate-buffered saline (PBS) at pH 7.4 for 20 min, followed by washing with PBS. Neurons underwent permeabilization using 0.5% Triton-X (Sigma-Aldrich, St. Louis, MO, USA) for 20 min on a shaker at RT. To block the background, neurons were treated with 2% goat serum and 0.1% Triton-X in PBS for 1 h at RT. Following three PBS washes, the cells were prepared for analysis through immunocytochemistry.

For confocal imaging, hippocampal neurons underwent overnight incubation at 4 °C with primary antibodies. Subsequently, the cells were washed using PBS and exposed to corresponding Alexa-conjugated secondary antibodies (Thermo Fisher, Waltham, MA, USA, 1:400) while covered with a thin foil for 1 h at RT. The following antibodies were used: anti-Gabra5 (NeuroMab, Davis, CA, USA, N52A/42, AB_2491075, 1:1000), anti-Gabra1 (Milipore, Burlington, MA, USA, AB5592, 1:1000), and anti-vGat (Synaptic Systems, Göttingen, Germany, 131-004, 1:1000). Images were taken utilizing a Nikon Eclipse Ti confocal laser scanning microscope (Nikon, Tokyo, 40× objective; NA 1.3) with NIS-Elements 4.30 software. The quantification of colocalization was performed with ImageJ (version: 1.54d) for Pearson correlation coefficient and Manders’ coefficient analysis via the JACop plugin (version: 2.1.1) [42].

## 3. Results

### 3.1. Composition of α5-Containing GABA_A_ Receptor from Hippocampus and Olfactory Bulb

**Figure 1:**
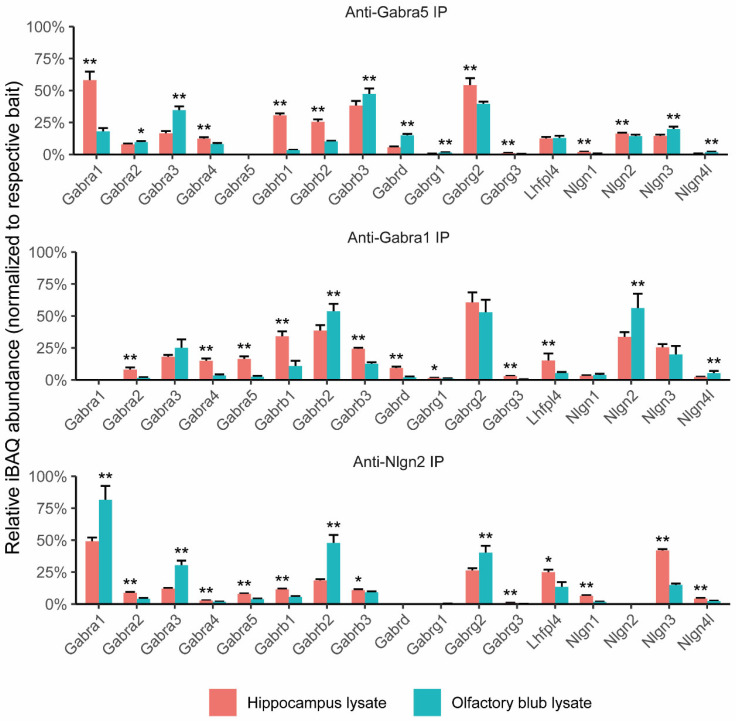
Quantitative proteomics of α5, α1, and Nlgn2 IPs performed on extracts of the hippocampus and olfactory bulb. The relative quantity of GABA_A_R subunits and known interactors identified from the anti-α5 IP (**upper panel**), anti-α1 IP (**middle panel**), and anti-Nlgn2 IP (**bottom panel**). IP experiments were performed with three biological replicates. The iBAQ abundance values per sample were normalized to the respective bait protein’s abundance, and linear regression was applied to compare relative prey abundances between brain regions. * *p* value < 0.05 and ** *p* value < 0.01.

To reveal the composition of the α5-containing GABA_A_ receptor from the hippocampus and olfactory bulb, we performed α5 subunit immunoprecipitations on both the hippocampus and olfactory bulb extracts followed by mass spectrometric analysis. The relative abundance of the GABA_A_R proteins normalized to α5 subunits from the IPs of the hippocampus and olfactory bulb are shown in Figure 1, upper panel. The iBAQ abundance values as a percentage of α5 and the raw intensity data of the GABA_A_R proteins are shown in Table S1. The complete information of identified proteins and peptides is provided in Data S1 and Data S2. An interesting finding was the promiscuous presence of different subunits including α1-4 subunits in the anti-α5 IPs from the hippocampal extract. In particular, α1 was most abundantly co-immunoprecipitated, which comprised 58.0% of the bait protein intensity. A second finding came from brain region differences. In the olfactory bulb, α5 IPs yielded lower amounts of α1 (18.0%) but showed higher abundances of α3 (34.7%), β3 (47.2%), and δ (15.0%) when compared to those from the hippocampus. In parallel, we included a negative control IP against the AMPAR receptor Gria2/3 subunit, which was shown not to pull down the GABA_A_R subunit (Chen et al., 2014). Using mass spectrometry to identify and quantify immunoprecipitated proteins from each IP, we found a low amount of GABA_A_ receptors in the anti-Gria2/3 IPs against the high abundance of known AMPA receptor subunits. This background GABR_A_A was of low intensity (<1% intensity in anti-GABA_A_R subunits’ IPs) and would not interfere with the quantitation of the pulldown GABR_A_A subunits (Table S2).

### 3.2. Composition of α1-Containing GABA_A_ Receptor from Hippocampus and Olfactory Bulb

While α1-4 were present in the α5 IPs, α1 was highly abundantly found in the hippocampus. To further interrogate their co-assembly, we performed anti-α1 IPs and mass spectrometry on both the hippocampus and olfactory extracts. In anti-Gabra1 IPs, we first demonstrated the co-IP of α1 and α5 (Figure 1, middle panel). Furthermore, we confirmed the differences in protein abundance between brain regions. The relative amount of α5 that co-immunoprecipitated with α1 was significantly higher in the IP performed on the hippocampus (16.3%) than in the one performed on the olfactory bulb (2.4%). Apart from α5, other GABA_A_ receptor subunits such as α2, α4, α5, β1, β2, β3, γ1, γ3, and δ demonstrated differences in relative abundance between the hippocampus and olfactory bulb. As for known interactors, Nlgn2 was found to be co-immunoprecipitated more abundantly in the olfactory bulb (56.2%), whereas the α1-GABA_A_R-co-immunoprecipitating Lhfpl4 was minor in the olfactory bulb (5.4%) compared with the one identified in the hippocampus (15.1%).

The α1 IP revealed differences in Nlgn2 quantities from the hippocampus and olfactory bulb. This difference was minor in the α5 IP. To validate these brain region differences, we performed IPs using anti-Nlgn2 antibodies (Figure 1, bottom panel). The most abundant protein from the Nlgn2 IP was α1, which together with β2 and γ2 form the main GABA_A_ receptor subtype in the brain. These subunits were more abundantly immunoprecipitated in the Nlgn2 IP performed on olfactory bulb lysate as compared to hippocampal lysate. This suggested that Nlgn2 shows a higher preference to associate with the α1β2γ2 GABA_A_ receptor in olfactory bulbs (Figure 1, middle panel).

### 3.3. Analysis of Hippocampal and Olfactory Bulb GABA_A_R Subcomplexes with Blue Native Gel Electrophoresis–Mass Spectrometry

We demonstrated that a higher proportion of α1-containing receptors interacted with Nlgn2 in the olfactory bulb as compared to the hippocampus (Figure 1). This also implicated that there were receptor subcomplexes that did not associate with Nlgn2. They most likely exist as naked receptors, as has been previously shown for the cerebellar α1-containing receptor [13,33]. To examine the GABA_A_R subcomplexes in more detail, we used BN-PAGE to separate protein complexes by their size and mass spectrometry to quantify and identify protein components within protein complexes. Nlgn2-associated GABA_A_Rs had higher mass than the naked receptors and therefore were visible as having higher molecular weight in the BN gel.

**Figure 2:**
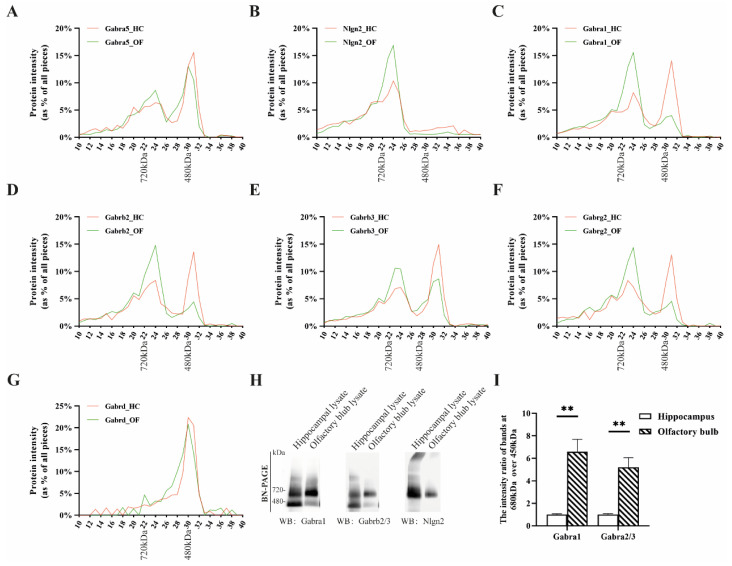
Migration of GABA_A_R subunits and known interactors obtained from hippocampus and olfactory bulb extracts with BN-PAGE and identified with mass spectrometry and immunoblotting. Migration profiles of the protein complexes containing (**A**) GABA_A_R α5, (**B**) Nlgn2, (**C**) GABA_A_R α1, (**D**) β2, (**E**) β3, (**F**) γ2, and (**G**) δ extracted from the hippocampus and olfactory bulb with BN-PAGE as identified with mass spectrometry. (**H**) Native GABA_A_R run on BN-PAGE from extracts of hippocampi and olfactory bulbs. The migration patterns of the receptors were visualized with immunoblots with antibodies against Gabra1, Gabrb2/3, and Nlgn2. (**I**) Quantitative analysis of the intensity ratios of bands at 680 kDa over 450 kDa on immunoblots. *n* = 3 samples from three independent regional brain whole tissue lysates; data are mean ± s.e.m. ** *p* < 0.01.

In line with the immunoprecipitation studies stated above, we first interrogated the protein abundance profiles of GABA_A_R α5 (Figure 2A). The α5-containing complexes predominantly migrated as two peaks in both the hippocampus and olfactory bulb, where the major peak migrated at ~450 kDa and the minor peak at ~680 kDa. Nlgn2 co-migrated with the receptors only at ~680 kDa (Figure 2B). The 450 kDa α5-containing protein complex detected here could be assigned to the naked form of the GABA_A_R, as the GABA_A_R alone was found to migrate ~450 kDa in the BN gel [33].

As α1 and α5 subunits were mutually co-immunoprecipitated (Table S2 and Figure 1), we further interrogated the protein abundance profiles of GABA_A_R α1. Figure 2C shows the naked and decorated forms of GABA_A_R α1 at 450 and 680 kDa, respectively, with large brain region differences in ratios between these two forms. In the hippocampus, most of the GABA_A_R α1 migrated at 450 kDa, and about 10% was observed at 680 kDa. In the olfactory bulb, this ratio was reversed with 60% of the total GABA_A_R α1 migrating at 680 kDa. Similar differences were also observed in β2-, β3-, and γ2-containing complexes (Figure 2D–F). This implied that the relative abundance of the Nlgn2-decorated form of α1/β2/γ2 GABA_A_R in the hippocampus was significantly lower than that of the olfactory bulb. The δ-containing complex was present at 450 kDa and showed no brain region difference (Figure 2G).

We then validated the proteomic findings with Western blotting. The hippocampus and olfactory bulb extracts were fractionated with BN-PAGE and immuno-stained for α1, β2/3, and Nlgn2 (Figure 2H). The intensity ratio of a 680 kDa immuno-stained band over a 450 kDa immuno-stained band in the olfactory bulb was significantly higher than that in the hippocampus (Figure 2I), confirming the higher abundance of Nlgn2-decorated α1/β GABA_A_R in the olfactory bulb. Together, these data were in line with our previously observed brain region differences in GABA_A_R forms (Figure 1).

### 3.4. The Subcellular Localization of the α1- and α5-Subunit-Containing Receptors in Cultured Hippocampus Neurons

**Figure 3:**
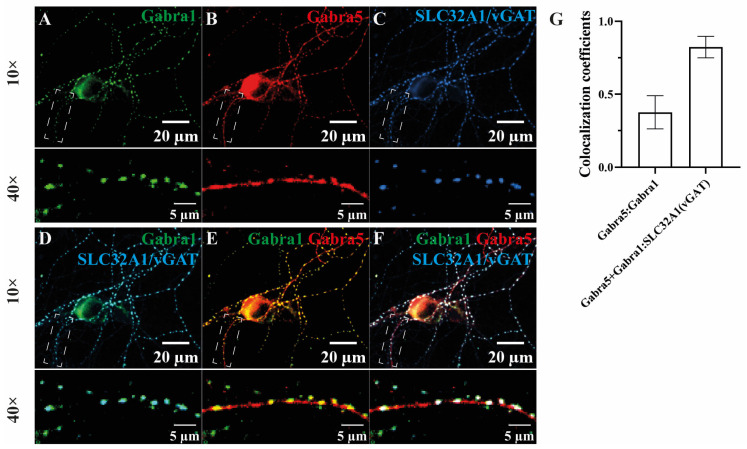
The α1-/α5-containing GABA_A_ receptors demonstrated synaptic localizations in a primary hippocampal neuronal culture. The distributions of α1 and α5 subunits were investigated (**A**,**B**) and both subunits were co-stained with the inhibitory presynaptic marker protein vGat (**C**). (**D**) The α1 subunit was mostly synaptically localized, opposing vGat, whereas α5 was distributed at both the soma and synapse and often showed co-localization with α1-vGat puncta at the synapse (**E**,**F**). (**G**) The Manders’ coefficient of the α1 subunit (green channel) with an α5 subunit signal (red channel) and colocalized α1 and α5 subunit signals (blue channel) were measured. The latter one is indicated by Manders’ coefficients with vGat; *n* = 15, fields of view; *n* = 3, cultures.

The co-precipitation of α1 and α5 subunits and the α5 subunit association with Nlgn2 may suggest the synaptic localization of these subunits in a single receptor type (Figure 1 and Figure 2, Table S2). We examined the subcellular distribution of α5 and α1 subunits at dendritic and synaptic sites using immunocytochemistry in a mouse hippocampal neuronal culture. The α1 subunit immunoreactivity showed synaptic localizations as puncta opposing the presynaptic marker protein vGat (SLC32A1) (Figure 3A,C,D), consistent with the previously reported predominant postsynaptic localization of the α1 subunit [33,43]. The α5 subunit exhibited a wider distribution of both synaptic and extra-synaptic localizations along the dendrite (Figure 3B), in agreement with previous studies [27,44,45,46]. An overlap of α1 and α5 immunoreactivity was found in many puncta (Figure 3E–G), supporting the existence of α1-/α5-containing receptors and their synaptic localizations.

## 4. Discussion

Our interaction proteomics study on the protein complexes of α1- and α5-containing GABA_A_R suggested brain region differences in GABA_A_R subunit composition between the hippocampus and olfactory bulb. First, the relative abundance of α1-/α5-containing GABA_A_R was higher in the hippocampus than in the olfactory bulb. Second, the α1-containing GABA_A_ receptor in the olfactory bulb was found to have a higher proportion that interacted with Nlgn2. In addition, using immunocytochemical staining, we demonstrated that α1-/α5-containing GABA_A_R was synaptically localized. Finally, we applied BN-PAGE-MS and immunoblotting on hippocampus and olfactory bulb lysate and confirmed that α1-containing GABA_A_Rs in the olfactory bulb showed more Nlgn2 protein interactions compared with the ones in the hippocampus.

Both IP and BN-PAGE are interaction–proteomics approaches to study protein complexes. One of the advantages of BN-PAGE is that it allows for the differentiation of subcomplexes incorporating the same protein or protein complex components. This separation cannot be achieved using IP only, since all the subcomplexes are simultaneously pulled down. In this study, we combined IP with specific antibodies and BN-PAGE to characterize the brain region differences in GABA_A_R protein complexes.

The α5-containing GABA_A_ receptor has been reported as being mainly expressed in the hippocampus and olfactory bulb [15,16,18,19], in which also the α1 subunit is highly expressed. Receptors containing the α5 subunit have been reported to distribute at both synaptic and non-synaptic sites in rat brains [45]. The α1-/α5-containing GABA_A_R has been previously identified in rat brains and further pharmacological studies on this type of receptor showed that it can display a lower binding affinity to benzodiazepine compared with α1 GABA_A_Rs [47].

GABA_A_-receptor-associated proteins have been found to play important roles in the regulation of GABA_A_R subunit assembly, delivery, and membrane insertion and anchoring, thereby modulating appropriate inhibitory neurotransmission and maintaining the excitation/inhibition balance in the brain [48,49].

Proteomic studies on GABA_A_ receptor complexes have been largely performed and focused on discovering novel GABA_A_-receptor-associated proteins [3,12,50,51]. However, few studies have been performed on brain region differences in the interaction between GABA_A_ receptors and well-known associated proteins. In this study, we also examined the relative abundance of GABA_A_ receptors interacting with Nlgn2 with BN-PAGE. Nlgn2 is a GABA_A_ receptor scaffold protein at the synapse and specifically interacts with the synaptic GABA_A_ receptor subtype, facilitating the synaptic clustering of GABA_A_ receptors [10,11]. Mutations in Nlgn2 genes, leading to impaired folding and trafficking of Nlgn2, have been found in autism subjects [52]. As previously reported, the Nlgn2-interacting and naked forms of the GABA_A_ receptor can be clearly differentiated by their molecular weight [33]. Surprisingly, we found that the Nlgn2-interacting GABA_A_ receptor was relatively more abundant in the olfactory bulb compared with the hippocampus, suggesting that the processing and regulation of sensory odor inputs requires a larger quantity of synaptic GABA_A_ receptors. This was supported by GABA_A_Rs that have been found to contribute to the odor-evoked inhibition of antennal lobe projection neurons in *Drosophila* on both fast and slow time scales [53]. However, the exact reason why GABA_A_Rs in olfactory bulbs more frequently interact with Nlgn2 and what physiological function(s) drive this will need future investigation.

## 5. Conclusions

In this study, based on a quantitative IP-MS analysis, we identified the existence of α1-/α5-containing GABA_A_R isoforms in mouse hippocampi and olfactory bulbs. A further quantitative analysis on this GABA_A_R isoform between the two brain regions revealed a significantly high expression level in the hippocampus. Immunocytochemistry on cultured hippocampus neurons revealed that the α1-/α5-containing GABA_A_Rs demonstrated synaptic localization. Moreover, the Nlgn2-interacting α1-containing GABA_A_Rs were also found to show brain region differences between mouse hippocampi and olfactory bulbs in a quantity that was more abundant than in olfactory bulbs.

## Data Availability

The raw data supporting the conclusions of this article will be made available by the authors, without undue reservation. The mass spectrometry proteomics data have been deposited to the ProteomeXchange Consortium via the PRIDE [54] partner repository with the dataset identifier PXD047550. All data will be made public once the manuscript is accepted for publication.

## Supplementary Materials

The following supporting information can be downloaded at: https://www.mdpi.com/article/10.3390/cells13010014/s1, Table S1: Anti-GABAAR α5 subunit IPs on mouse hippocampus and olfactory bulb; Table S2: Overview of all IPs_only GABAAR and AMPAR selected; Data S1: Protein and peptides information from IP_batch 1. Data S2: Protein and peptides information from IP_batch 2.
